# Metabolism and bioavailability of newly developed dietary fiber materials, resistant glucan and hydrogenated resistant glucan, in rats and humans

**DOI:** 10.1186/s12986-016-0073-2

**Published:** 2016-02-16

**Authors:** Sadako Nakamura, Kenichi Tanabe, Shigeki Morita, Norihisa Hamaguchi, Fumio Shimura, Tsuneyuki Oku

**Affiliations:** Institute of Food, Nutrition and Health, Jumonji University, 2-1-28, Sugasawa, Niiza, Saitama 352-8510 Japan; Graduate School of Human Health Science, University of Nagasaki Siebold, 1-1-1, Manabino, , Nagayo, Nagasaki 851-2195 Japan; Department of Food Science and Nutrition, Nagoya Women’s University, 3-40, Shioji, Mizuho-ku, Nagoya 467-8610 Japan; Institute of Food Materials, Nippon Shokuhin Kako Co. Ltd., Tajima, Fuji, 417-8530 Japan

**Keywords:** Metabolism, Bioavailability, Available energy, Dietary fiber materials, Resistant glucan, Hydrogenated resistant glucan

## Abstract

**Background:**

Resistant glucan (RG) and hydrogenated resistant glucan (HRG) are new dietary fiber materials developed to decrease the risk of metabolic syndrome and lifestyle-related diseases. We investigated the metabolism and bioavailability of RG and HRG using rats and humans.

**Methods:**

Purified RG and HRG were used as test substances. After 25 Wistar male rats (270 g) were fed with an experimental diet (AIN93M diet with the cellulose replaced by β-corn starch) *ad libitum* for 1 week, they were used for the experiment involving blood collection and circulating air collection. Ten participants (5 males, 22.5 y, BMI 20.4 kg/m^2^; 5 females, 25.8 y, BMI 20.9 kg/m^2^) voluntarily participated in this study. The study was carried out using a within-subject, repeated measures design. Effects of RG and HRG on the response for blood glucose and insulin and hydrogen excretion were compared with those of glucose and a typical nondigestible and fermentable fructooligosaccharide (FOS) in rats and humans. Available energy was evaluated using the fermentability based on breath hydrogen excretion.

**Results:**

When purified RG or HRG (400 mg) was administered orally to rats, blood glucose and insulin increased slightly, but less than when glucose was administration (*P* < 0.05). Hydrogen started to be excreted 120 min after administration of RG with negligibly small peak at 180 min, thereafter excreted scarcely until 1440 min. Hydrogen excretion after HRG administration showed a larger peak than RG at 180 min, but was markedly less than FOS. RG and HRG were excreted in feces, but not urine. When purified RG or HRG (30 g) were ingested by healthy humans, blood glucose and insulin levels increased scarcely. Breath hydrogen excretion increased slightly, but remarkably less than FOS. Ingestion of purified RG or HRG (5 g) to evaluate available energy, increased scarcely glucose and insulin levels and breath hydrogen excretion. Available energy was evaluated as 0 kcal/g for purified RG and 1 kcal/g for HRG.

**Conclusion:**

The bioavailability was very low in both humans and rats, because oligosaccharide of minor component in purified RG and HRG was metabolized via intestinal microbes but major components with higher molecular weight were metabolized scarcely. Moreover, the ingestion of 30 g of RG or HRG did not induce apparent acute side effects in healthy adults. RG and HRG might potentially be used as new dietary fiber materials with low energy.

## Background

Resistant glucan (RG) and hydrogenated resistant glucan (HRG) are new dietary fiber materials that have been developed to decrease the risk of metabolic syndrome and lifestyle-related diseases. RG is prepared by heating glucose at 180 °C in the presence of activated carbon [1], and is a mixture of monosaccharides, oligosaccharides and glucose polymers with an average molecular weight of approximately 2600. RG is a white odourless powder that is easily dissolved in cold water and can be widely used as a low energy bulking carbohydrate material. HRG is produced from RG by hydrogenation, and is more stable than RG toward degradation by the Maillard reaction [1].

In order to use widely RG and HRG as food materials, it is necessary to clarify the physiological effects of RG and HRG on the response for blood glucose and insulin, the occurrence of abdominal symptoms, the maximum non-effective dose for osmotic diarrhea, fermentability by intestinal microbes and metabolic fate. In addition, the available energy including nondigestible carbohydrates must be expressed as nutritional labeling in processed foods based on the Law of some countries [2, 3]. We have already proposed the indirect and simple method to evaluate the available energy of dietary fiber materials in healthy humans [4–6]. In accordance with our method, the digestibility is estimated based on the response of blood glucose and insulin and the fermentability is estimated by breath hydrogen excretion. In the present study, the metabolism and bioavailability of RG and HRG were investigated using rats and healthy human subjects and the available energy for purified RG and HRG was estimated by our proposed method.

In a previous study [7], it has been demonstrated that purified RG and HRG were consist of small amounts of glucose and digestible and fermentable oligosaccharides, and large amounts of nondigestible glucose polymers with high molecular weight. Purified RG and HRG are hydrolyzed to a limited extent by digestive enzymes, and did not inhibit small intestinal disaccharidases such as sucrase and maltase. These results agree roughly with the dietary fiber content of purified RG (99 %) and HRG (82 %), which have been measured by the enzymatic-high performance liquid chromatography (HPLC) method of the Association of Official Analytical Chemists (AOAC) 2001.03 method [8].

In rats, the ingestion of a diet containing 5 % of purified RG or HRG caused loose stools in consecutive feeding experiments. However, the growth and development of the rats were normal and no specific abnormal observations in the tissues and organs were inspected, except for a notable increase in the weight of cecal tissue and content. In addition, blood biochemical parameters such as glucose, insulin, total cholesterol, free fatty acid, total protein, AST and ALT *etc.* were normal in the RG, HRG, resistant maltodexitrin (RMD), fructoologosaccharide (FOS) and control groups and they were not significantly different among the five groups [7]. Furthermore, the abnormality is not detected in acute, subacute and chronic toxicity tests of RG [9]. Also, RG and HRG have been confirmed in the earlier work on the safety and stability of RG [1]. Therefore, RG and HRG seem to be safety as nondigestible carbohydrate materials.

RMD and polydextrose (PD) have been widely used in the processed foods and the health claim for its use in processed foods is potentially reduce the energy density [10–14]. The available energy is evaluated as 1 kcal/g for RMD and PD based on our proposed method [5]. Also, Auerbach, et al. [12] and Goda, et al. [14] report that the available energy is estimated as 1 kcal/g each for RMD and PD using different methods. The results obtained from in vitro and in vivo experiments for RG and HRG demonstrate that RG and HRG are notably resistant to hydrolysis by human α-amylase and small intestinal enzymes and are difficult to be fermented by intestinal microbes compared with RMD and PD [7]. Therefore, we hypothesize that the available energy of purified RG and HRG is less or at least the same than quality obtained from RMD and PD.

In the present study, prior to the human study, we performed a study using rats to investigate some physiological effects such as the response indicated by blood glucose and insulin, hydrogen excretion, and fecal and urinary excretion after single dose of the tested substances. Then, we carried out human experiments using a within-subject repeated measures design. The response of blood glucose and insulin, breath hydrogen excretion, the occurrence of abdominal symptoms, and fecal shape were investigated using healthy human subjects. In addition, the available energy of purified RG and HRG for human subjects was evaluated by an indirect and simple method based on fermentability using breath hydrogen excretion [4–6, 15, 16]. The results obtained will contribute to the practical application of RG and HRG for potentially decreasing the risk of metabolic syndrome and lifestyle-related diseases.

## Methods

### Ethical approval of the study protocol

All experiments complied with the code of ethics of the World Medical Association (Declaration of Helsinki, Oct. 2013). The study protocol involving human subjects was approved by the Ethical Committee of the University of Nagasaki (received No.184, approval No.177). All participants provided written informed consent to participate in the study.

The protocol for animal studies was approved by (approved No. 2011-15) the Committee on Animal Experiments of the University of Nagasaki. These experiments were conducted according to the Guidelines on the Care and Use of Laboratory Animals (National Institutes of Health, MD, USA) and the standards relating to the Care and Management of Experimental Animals (Notification number 88, from the Prime Minister’s Office). All experiments were carried out in the Public Health Nutrition Laboratory of the Graduate School of Human Health Science, University of Nagasaki Siebold.

### Materials

Purified RG (Nihon Shokuhin Kako Co., Ltd., Tokyo, Japan) by removing mono- and di-saccharides from RG, was used to focus for the main components with degrees of polymerization (DP) of more than 2 in the present study, and HRG (Nihon Shokuhin Kako Co., Ltd.) was also used to compare with purified RG. RMD (Fibersol-2, dietary fiber content: 92 %, Matsutani Chemical Industry Co., Ltd., Hyogo, Japan), FOS (purity: > 99 %, Meiji Co., Ltd., formerly-Meiji Seika Kaisha Ltd., Tokyo, Japan) and glucose (Nihon Shokuhin Kako Co., Ltd.) were also used in the present study. Glucose was used as a reference of the response of blood glucose and insulin. RMD is a mixture of monosaccharides, oligosaccharides and glucose polymers with an average molecular weight of about 2000 [10, 11]. It was used as a reference of dietary fiber established already. FOS was used as a reference of a typical oligosaccharide that is not hydrolyzed by digestive enzymes and is fermented completely by intestinal microbes [17, 18] and consists of 1-kestose (39 %), nystose (53 %), and 1^F^-β-fructofranosyl-nystose (7 %).

### Animal experiments using rats

#### Animals and diets

To investigate the effect of RG and HRG on the response of blood glucose and insulin and the fermentability of RG and HRG by intestinal microbes, 25 Wistar male rats (270 g, Clea Japan Inc., Tokyo, Japan) as the previous studies [7, 17], were fed with an experimental diet *ad libitum* or 1 week. They were used twice: for the experiment involving blood collection and the experiment involving circulating air collection. The experimental diet was AIN93M diet [19] with the cellulose replaced by β-corn starch to exclude the effect of cellulose on the intestinal microflora. Rats were housed in individual stainless steel cages and kept at room temperature (22–24 °C). The relative humidity was maintained at 50–55 % with a 12-h light/dark cycle.

### Experimental setup for determination of plasma glucose and insulin responses

The effects of glucose, RG, HRG and RMD on the response of blood glucose and insulin were investigated. Glucose was used as a control and RMD was used as a positive control, as it has been established previously as a dietary fiber material. The dose (400 mg) of test substance which does not cause loose stool in rat was dissolved in 2.5 ml of distilled water as the previous studies [18, 20]. The test solution was orally administered to a rat using a stomach tube after fasting for 16 h (*n* = 5 per group). Blood (120 μl) was collected from the rat’s tail vein using heparinized hematocrit tubes at 30-min intervals until 180 min after administration of the test substance, and then the plasma was prepared by hematocrit centrifugation (3300, Kubota Co., Ltd., Tokyo, Japan) at 2000 × g, 20 °C for 5 min.

Glucose concentration was measured by the Trinder method using glucose oxidase [21]. After plasma (10 μl) was pipetted into a small test tube, glucose oxidase reagent (1.5 ml) was added and the test tube was incubated at 37 °C for 15 min. Then the test tube was steeped in boiling water for 150 s to stop the reaction. The absorbance was read immediately using a spectrophotometer (UVmini-1240, Shimadzu Corp., Kyoto, Japan) at a wavelength of 505 nm. The insulin concentration was measured immunologically by an ELISA kit (Morinaga Biochemical Institute Co., Kanagawa, Japan) using guinea pig-derived antibody [22].

### Circulating air collection and analysis of hydrogen

**Fig. 1:**
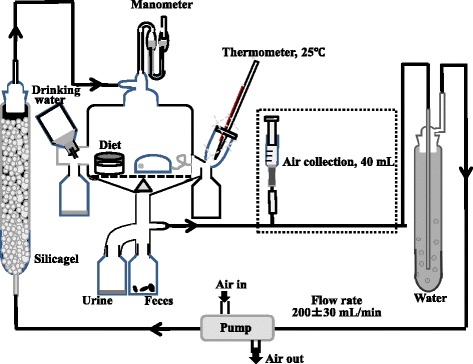
Metabolica apparatus to collect the circulating air, urine and faeces of rats

The effects of FOS, RG, HRG and RMD on hydrogen excretion were also investigated using rats. FOS was used as a reference of typical nondigestible and fermentable oligosaccharide to compare fermentability. Each test solution was prepared as 400 mg in 2.5 ml distilled water. Five rats fed with the experimental diet and 15 rats fed with the experimental diet for 2 days after blood collection, were used in the experiment involving circulating air collection. Fifteen rats which had been used in the experiment of blood collection were administered with same test solution as the experiment of blood collection and five new rats were administered with FOS solution. The test solution (400 mg/2.5 ml) was orally administered to a rat (*n* = 5 per group) after fasting for 16 h. Then the rats were immediately moved to the Metabolica apparatus (5-gang, Sugiyamagen Co., Ltd., Tokyo, Japan) (Fig. 1) to collect the circulating air for hydrogen determination.

The flow rate of the circulating air in the apparatus was adjusted to 200 ± 30 ml/min. To obtain the initial value, 40 ml of circulating air was collected using a plastic syringe at 10 min after moving the rat into the apparatus. Then the circulating air was collected at 1-h intervals until 4 h, 2-h intervals from 4 h to 10 h, and at 24 h after administration of the test solution [23]. To avoid hunger, the experimental diet was fed to the rats at 4 h after administration of test solution. Five millilitres of the circulating air were loaded into a simple gas chromatograph (Breath Gas Analyzer BGA1000D, Laboratory for Expiration Biochemistry Nourishment Metabolism Co., Ltd., Nara, Japan) to measure the concentration of hydrogen.

### Extraction of RG and HRG from feces and urine and analysis by HPLC

After RG or HRG (400 mg/2.5 ml) was orally administered to the rats, their urine and feces were collected separately using the Metabolica apparatus for 24 h. The collected feces were combined, stood for 10 min in boiling water to stop the reaction by microbes-derived enzymes and homogenized with 9 volumes of ice-cold 0.9 % NaCl using a homogenizer (Polytron; Kinematica Inc., Lucerne, Switzerland). Then the suspension was centrifuged under refrigeration (Himac CR22GII, Hitachikoki Co., Ltd., Tokyo, Japan) at 20,000 × g, 4 °C for 20 min. The precipitate was re-suspended with 4 volumes of 0.9 % NaCl and centrifuged again. The supernatants collected were combined and filtered through a membrane filter (0.22 μm × 13 mm; Millipore Corp., MA, USA) to remove bacteria and small pellets, then used in the HPLC analysis. The urine collected was measured the volume, and then was also filtered through a membrane filter (0.22 μm × 13 mm) to remove bacteria and small pellets.

The analysis of RG and HRG was carried out using an HPLC system (LC-20 AD; Shimadzu Corp.) using a refractive index detector (RID-10A; Shimadzu Corp.) and a Shodex SUGAR KS-802 column (8.0 φ × 300 mm; Showa Denko Co., Ltd., Tokyo, Japan). Analysis was performed at a column temperature of 70 °C. The sample (10 μl) was injected into the HPLC system and eluted with deionized distilled water at a flow rate of 0.5 ml/min [24]. The limit concentration of detection of glucose by HPLC analysis was 10 μg/ml.

### Human experiments using healthy subjects

#### Participants

The present study involving human participants was carried out using a within-subject, repeated measures design. Each subject ingested repeatedly the test substance at intervals of 1-week or more. Ten healthy participants (5 males, 22.5 ± 1.7 year old, 59.7 ± 2.5 kg, BMI 20.4 ± 1.2 kg/m^2^; 5 females, 25.8 ± 3.8 year old, 50.5 ± 4.0 kg, BMI 20.9 ± 1.1 kg/m^2^) voluntarily participated in this study. The exclusion criteria were a history of gastrointestinal diseases, carbohydrate malabsorption, diabetes, obesity or pulmonary disease. The average value of fasting blood glucose concentration of the subjects was 4.6 ± 0.2 mmol/L and it was within normal range. Before the experiments, we ensured that all participants were hydrogen producers and not methane producers. The participants had not taken any antibiotics or laxatives in the 2 weeks before the experiments.

### Preparation of test solutions

The effects of glucose, FOS, RG and HRG on blood glucose and insulin response and breath hydrogen excretion were investigated. Glucose was used as a control and FOS was used as a typical nondigestible and fermentable oligosaccharide. Thirty grams of glucose, RG or HRG were dissolved in 120 ml of warmed safety water as the previous studies [4, 5, 25–28]. The tolerance level of FOS, because of its osmotic effects in the large bowel, is 0.3 g/kg of body weight for Japanese normal people [6, 25]. Therefore, 5 g of FOS was dissolved in 120 ml of warmed safety water in accordance with our proposed method [4, 5, 25]. Five grams of RG or HRG were also dissolved in 120 ml of warmed safety water to evaluate available energy.

### Special meals for experiments

The period during which the food intake of the subjects was restricted was very long. Hence, the experimental meals, from which hydrogen was not produced, were given to the subjects after the final blood collection, at 7 h and 11 h after ingestion of test substance. Canned tuna fish (Sea-chicken mild, 80 g, Hagoromo Food Co., Ltd., Shizuoka, Japan), boiled egg (50 g) and tea containing 10 g of table sugar were used as food materials for experimental meals. The energy supplied to the subjects on the experimental day was 895 ~ 970 kcal and protein was 58 ~ 65 g. If needed, tea containing sugars or boiled egg was given. In addition, each subject was given a multivitamin tablet containing mineral (Nature Made, Otsuka Pharmaceutical Co., Ltd., Tokyo, Japan) as a supplement every day during the experimental periods.

### Experimental protocol

All test substances were given in a random order at intervals of 1-week or more. Experiments were carried out under the direction of a physician. The experimental protocol was carried out in accordance with the methods employed in our previous studies [4–6, 25–29].

After overnight fasting, the subjects came to our laboratory in the morning, their health status was examined and their blood pressure and pulse rate were measured. Then, 750 ml of end-expiratory gas were collected using a collection bag (Quin Tron Instrument Co., Inc., WI, USA) and 120 μl of blood from the fingertip using a heparinized hematocrit tube. After ingesting the test substance, the end-expiratory gas was collected at 1-h intervals until 14 h [4], and blood (120 μl) was collected at 30-min intervals until 180 min. Participants were prohibited from ingesting foods containing nondigestible carbohydrates for 3 days before each experimental day. During the experiment, they were also prohibited from ingesting foods or beverages except for warmed water and tea, as well as from sleeping and smoking. Subjects were required to sit on a chair and were prohibited from exercising with hyperventilation until the final collection of end-respiratory gas. Participants ingested an experimental meal that did not produce breath hydrogen, so that they would not feel hungry.

After ingesting the test substance, the type and onset time of abdominal symptoms, the frequency of defecation and the macroscopic observation of stool shape were recorded by the participants themselves using constructed format. The research supervisor explained to all participants the method for self-recording and classifying the stool shape and abdominal symptoms, and then confirmed their recordings, when they were received. The examination of stools used the following descriptors: stage 1, very hard; stage 2, hard; stage 3, normal; stage 4, soft; stage 5, very soft; and stage 6, watery [30]. The questionnaire on abdominal symptoms were asked to the subjects about their experience of upper and lower abdominal pain, vomiting, nausea, thirst, flatus, distension, and borborygmus for 14 h after ingesting the test substance.

### Assay of plasma glucose and insulin and analysis of breath hydrogen

The plasma glucose and insulin concentrations and hydrogen in the end-expiratory gas were measured by the methods described above.

### Evaluation of relative available energy of RG and HRG

An indication of energy content is an essential item for the nutritional labelling of processed foods [2, 3]. The available energy in dietary fiber materials and nondigestible oligosaccharides can be evaluated using an indirect and simple method based on the breath hydrogen excretion which reflects the fermentability of nondigestible carbohydrate [4–6, 25]. As energy production is dependent on the fermentability of nondigestible carbohydrates, the available energy can be categorized to 0 kcal/g, 1 kcal/g and 2 kcal/g. Available energy was calculated based on the ratio of fermentability versus FOS, which has 2 kcal/g of available energy, in the present study [15, 16].

To evaluate the available energy of RG and HRG using the indirect and simple method based on fermentability, 5 g of the test substance were ingested by a healthy subject as well as other dietary fiber materials. The end-respiratory gas was collected to measure hydrogen excretion for 14 h after ingestion. FOS (2 kcal/g) was used as a convenient reference to estimate the relative fermentability [4, 5, 25].

### Calculations and Statistical analyses

We calculated the means and standard deviations in blood glucose, insulin and concentration of hydrogen excretion in rats and humans. Evaluation of the available energy in humans was calculated based on the ratio [4, 5]. The ratio was calculated based on the areas under the curve (AUC) of hydrogen excretion by the ingestion of test materials versus that of FOS. Thereafter, the available energy was classified into 0 kcal/g, 1 kcal/g and 2 kcal/g as described above. The normal distribution was tested, and the data with normal distribution were compared with control (reference) using ANOVA and Dunnett’s *post hoc* test or Tukey’s *post hoc* test, and non-normal distribution data were compared using non-parametric analysis by Steel or Steel-Dwass method. A *P*-value less than 0.05 obtained two-sided analysis was considered as significant using SPSS for Windows 20.0 (SPSS Inc., Tokyo, Japan).

## Results

### Animal experiments using rats

#### The effects of RG and HRG administration on blood glucose and insulin levels

**Fig. 2:**
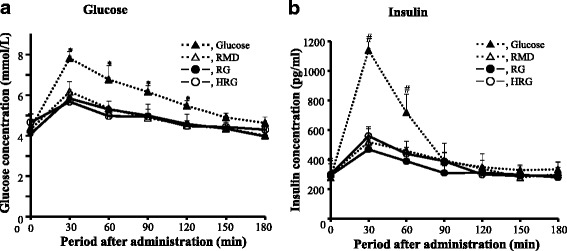
Changes of blood glucose and insulin levels after oral administration of RG, HRG or RMD to rats fed with an experimental diet. RG, resistant glucan; HRG, hydrogenated resistant glucan; RMD, resistant maltodextrin. Test substance (400 mg/2.5 ml) was administered orally to each rat which was fed with an experimental diet (n = 5 per group) and blood was collected from the tail vein at indicated periods. Values were means ± SD. *,#, The elevation of glucose in RG, HRG, and RMD were significantly lower than that of glucose at each time point, at *P* < 0.05 by Dunnett’s *post hoc* test. There was no significantly difference among RG, HRG, and RMD by Tukey’s *post hoc* test

The plasma glucose and insulin concentrations increased spontaneously at 30 min after glucose (400 mg/2.5 ml) was administered orally to rats (Fig. 2a, b). The results show that the response of blood glucose and insulin is normal in rats. For RG administration, the plasma glucose level increased to 5.6 mmol/L and plasma insulin level reached at more than 450 pg/mL at 30 min after administration. The response of plasma glucose and insulin after administration of HRG and RMD was similar to that of RG and not significantly different among three test substances. Moreover, the response of plasma glucose and insulin after administration of RG, HRG and RMD were significantly lower than those of the administration of glucose (*P* < 0.05). These results suggest that when RG and HRG are given orally to rats, they are partially hydrolyzed by gastrointestinal enzymes and cause similar increases in blood glucose and insulin levels.

### The effects of RG and HRG administration on hydrogen excretion

**Fig. 3:**
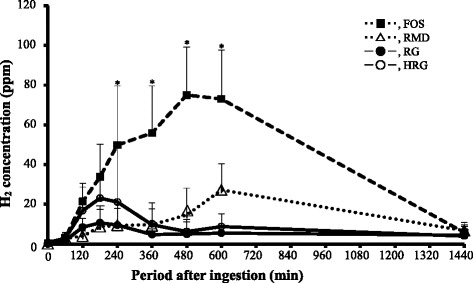
Changes of hydrogen excretion after oral administration of RG HRG or RMD to rats fed with an experimental diet. FOS, fructooligosaccharide; RG, resistant glucan; HRG, hydrogenated resistant glucan; RMD, resistant maltodextrin. Immediately after test substance (400 mg/2.5 ml) was orally administered to each rat fed with an experimental diet (*n* = 5 per group), rats were moved to the Metabolica apparatus to collect circulating air. Values were means ± SD. *, The excretion of hydrogen was significantly lower in RG, HRG and RMD than in FOS at each peak point, at *P* < 0.05 by Steel method. There was no significant difference among RG, HRG, and RMD by Steel-Dwass method

We considered that the excretion profile of hydrogen was normal, because when FOS (400 mg/2.5 ml) was given to rats, hydrogen which is produced only through fermentation by intestinal microbes, started to excrete about 120 min after administration and reached the maximum (80 ppm) at 480 min (8 h) (Fig. 3). For purified RG (400 mg/2.5 ml), hydrogen was excreted less than 10 ppm about 120 min after administration and then decreased to the basal level at 360 min. Hydrogen excretion after HRG administration (400 mg/2.5 ml) exhibited a similar behaviour to that of RG and was not significantly different. Hydrogen excretions of RG and HRG were significantly lower than that of FOS (*P* < 0.05). Hydrogen excretion after RMD administration showed a different pattern from that of RG and HRG, and it was significantly lower compared with that of FOS (*P* < 0.05).

### Urinary and fecal excretion of RG and HRG administered to rats fed with control diet

After RG or HRG (400 mg/2.5 ml) was orally administered to rats fed with the experimental diet, urine and feces were separately collected with the Metabolica apparatus for 24 h. Neither RG nor HRG were detected in the urine, but 20.4 % of the RG and 23.2 % of the HRG administered were excreted into feces for 24 h.

### Human experiments using healthy subjects

#### Subject participation

No participants dropped out of the study and experienced side effects. Their health status throughout the entire study period was well. The average intake of energy by a participant was 935 kcal/day and that of protein was 61 g/day.

### The effects of ingestion of RG and HRG on blood glucose and insulin levels and breath hydrogen excretion

**Fig. 4:**
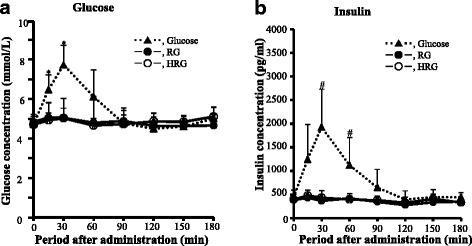
Change of plasma glucose and insulin levels after ingestion of RG or HRG in healthy human subjects. RG, resistant glucan; HRG, hydrogenated resistant glucan. After overnight fasting, 30 g of test substance (glucose, Purified RG and HRG) were ingested by 10 healthy subjects and blood (120 μl) was collected from the tip of finger at 30 min-intervals for 180 min after ingestion. Values were means ± SD. *, #, Plasma glucose (**a**) and insulin (**b**) concentrations in RG or HRG administration were significantly lower than those in glucose administration at each peak point, at *P* < 0.05 by Dunnett’s *post hoc* test for glucose (**a**) and by Steel method for insulin (**b**)

Figure 4 shows the changes in blood glucose and insulin levels after the ingestion of 30 g of glucose, purified RG or HRG by healthy participants. As the incremental blood glucose concentration was approximately 3.1 mmol/L at 30 min after the ingestion of glucose, the participants were normally tolerant of glucose. After ingestion of RG or HRG, both the blood glucose and insulin concentrations showed no incremental response. Blood glucose and insulin concentrations in RG ingestion were significantly lower than that of glucose administration at 30 min after ingestion (*P* < 0.05). Also, blood glucose and insulin concentrations in HRG ingestion were distinctly lower than that of glucose administration at 30 min as well as RG. These results which blood glucose and insulin concentrations increase scarcely after ingestion of RG or HRG are distinctly different from the results observed in rats.

**Fig. 5:**
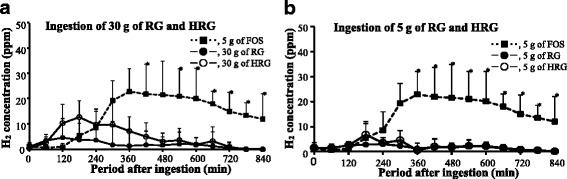
Changes of breath hydrogen excretion after ingestion of RG or HRG in healthy human subjects. FOS, fructooligosaccharide; RG, resistant glucan; HRG, hydrogenated resistant glucan. Test solution containing 30 g of purified RG or HRG was ingested by 10 healthy subjects shown in Fig. 4 after overnight fasting, and end-respiratory gas (750 ml) was collected using collection bag at 1-h intervals for 14 h after ingestion (**a**). Furthermore, to evaluate the available energy test solution containing 5 g of purified RG or HRG was ingested by healthy subjects after overnight fasting (**b**). FOS (5 g) was used as a typical oligosaccharide which is nondigestible and completely fermentable. Figure 5-a shows the change of hydrogen excretion 14 h after ingestion of 30 g of test substance, and Fig. 5-b shows the change of hydrogen excretion 14 h after ingestion of 5 g of test substance. Values were means ± SD. *, The excretion of breath hydrogen in RG or HRG ingestion were significantly lower versus that in FOS ingestion, at *P* < 0.05 by Steel method. There was no significantly difference between RG and HRG by Steel-Dwass method

The participants enrolled in the present study, were normal responders to breath gas test. When FOS (5 g), which is not digested by intestinal enzymes and is completely fermented by intestinal microbes, was ingested by healthy participants, no increasing on blood glucose and insulin concentration was observed (data not shown). The breath hydrogen started to excrete at 3 h after ingestion and showed a peak (23 ± 9 ppm) at 6 h after administration (Fig. 5a). Thereafter, it decreased gradually, but continued to excrete over 14 h until the end of the experiment. In the ingestion of purified RG (30 g), breath hydrogen started to excrete slightly at 1 h after ingestion, and then showed a flat without a clear peak during 2-4 h after ingestion. Thereafter, hydrogen was excreted scarcely up to the end of the experiment (Fig. 5a). Although breath hydrogen from ingesting HRG (30 g) started to excrete clearly about 2 h later than that from RG, but it was not significantly different.

In the experiments to evaluate the available energy, hydrogen excretion after ingesting of RG (5 g) did not increase and showed a flat during the experiment. Also, hydrogen excretion after HRG ingestion (5 g) was less than 5 ppm at 3 h after ingestion and were markedly smaller compared with a peak value (23 ppm) at 6 h after FOS ingestion (*P* < 0.05) (Fig. 5b). Namely, hydrogen excretion after ingestion of RH or HRG (5 g) was very little and did not show a significant increment.

Table 1 shows a comparison of the AUC from the excretion curves of breath hydrogen for 14 h after ingestion of the test substances (5 g or 30 g). Although breath hydrogen excretion was not 0 ppm at 0 min after ingestion (5 g) of FOS, RG or HRG, the AUC from the curve of breath hydrogen excretion for 14 h after ingestion was calculated simply. AUC versus time (14 h) was 850 ppm after purified RG (5 g) ingestion, 1250 ppm after HRG (5 g) ingestion and 11,740 ppm after FOS (5 g) ingestion. AUC versus time (14 h) of high level dose (30 g) was 1320 ppm for purified RG and 3150 ppm for HRG, respectively, and the AUC values were clearly larger than that of lower level dose.

**Table 1:** Estimation of available energy of RG and HRG in healthy human

	Nondigestable fractions			Digestable fractions		Estimated available energy (kcal/g)
	AUC (ppm/14 h)	Ratio versus AUC of FOS (%)	Calculated energy (kcal/g)	Content (%)	Calculated energy (kcal/g)	Calculated actual
			(1)		(2)	(1) + (2)
FOS	10,200 (7,800–15,855)	100.0	2			2
RG	601 (120–1,151)	6.1	0.12	1	0.04	0.16 → 0
HRG	674 (467–2,500)	6.6	0.13	18	0.72	0.85 → 1

*FOS* fructooligosaccharide, *RG* resistant glucan, *HRG* hydrogenated resistant glucan, *AUC* area under the curve. Five grams of test substances were ingested by healthy subjects. Data were expressed as median and interquartile in 10 healthy subjects. AUCs versus time for 14 h after ingestion of test substance were calculated based on the date shown in Fig. 5. The available energy of digestable fractions was calculated by content (%) × 4 kcal/g. Available energy was calculated based on our proposed method [4–6]

### Evaluation trial of available energy of RG and HRG

To evaluate the available energy, 5 g of purified RG or HRG were ingested by healthy participants as well as the evaluation of other dietary fiber materials [4–6]. Blood glucose and insulin concentrations did not increase overall and breath hydrogen excretion was negligible after purified RG ingestion and increased very slightly after HRG ingestion (Fig. 5b). The AUC from the curve of breath hydrogen excretion for 14 h after ingestion was 11,740 ppm for FOS ingestion, 850 ppm for purified RG ingestion and 1250 ppm for HRG ingestion (Table 1). Based on the available energy of FOS is 2 kcal/g, that of RG was evaluated as 0.14 kcal/g (850 ÷ 11,740 × 2 = 0.14) and that of HRG was evaluated as 0.21 kcal/g (1250 ÷ 11,740 × 2 = 0.21). The content of dietary fiber, which is resistant for hydrolyzing enzymes in AOAC 2001.03 method, are 99 % for purified RG and 82 % for HRG, respectively. Thus, the content of digestible saccharide, which the available energy is 4 kcal/g, is 1 % (0.04 kcal/g) for purified RG and 18 % (0.72 kcal/g) for HRG. Therefore, the total available energy becomes to 0.18 kcal/g (0.14 + 0.04 = 0.18) for purified RG and 0.93 kcal/g (0.21 + 0.72 = 0.93) for HRG, respectively. Because numerical values to two decimal places are not practicable for use in nutrition education and nutritional labelling, the nearest whole numbers are used [15, 16]. Therefore, the relative available energy values were 0 kcal/g for purified RG and 1 kcal/g for HRG.

## Discussion

In the present study we investigated the metabolism and bioavailability of RG and HRG using rats and healthy human subjects, thereafter we estimated that the available energy was 0 kcal/g for purified RG and 1 kcal/g for HRG in healthy humans, respectively. A small amount of oligosaccharides in RG and HRG were only digested and fermented slightly, while a large amount of high molecular weight carbohydrates were digested scarcely and fermented barely in healthy humans. These results demonstrate that the bioavailability of purified RG and HRG is very low in both humans and rats, although the response of blood glucose and insulin and the breath hydrogen excretion are different a little between rats and humans. Therefore, we discussed first in terms of rats, and next from the view point of the bioavailability in humans.

### Animal experiments using rats

When purified RG or HRG (400 mg/2.5 ml) was orally administered to rats, the blood glucose and insulin concentrations showed a small but clear peak at 30 min after administration, although the elevation was clearly lower than that of glucose administration. Previous experiments have demonstrated that purified RG and HRG contain a small amount of glucose and a small amount of oligosaccharides which are hydrolyzed by the small intestinal enzymes [7]. Therefore, the elevation of blood glucose level after the administration of purified RG or HRG seems to depend on a small amount of glucose and digestible oligosaccharides. Although the contents of glucose and digestible oligosaccharide were higher in HRG than in purified RG, the elevation of blood glucose and insulin was not significantly different between purified RG and HRG in rats. The blood glucose and insulin levels might be unable to respond because the amount of glucose and digestible oligosaccharide in the purified RG and HRG administered was very little. The blood glucose level was elevated by the administration of RMD as well as RG and HRG. RMD also contains a small amount of glucose and digestible oligosaccharide [10]. Dietary fiber materials that are not natural and have an average molecular weight of 1500-3000 seem to elevate slightly blood glucose and insulin levels. Although it has been recognized that natural dietary fiber materials do not increase blood glucose and insulin levels, the newly developed materials which contain a small amount of glucose and digestible oligosaccharides seems to increase slightly blood glucose and insulin levels.

Hydrogen is a specific product of fermentation of nondigestible carbohydrate by intestinal microbes. The amount of hydrogen excretion fluctuates widely from rat to rat and among groups, and includes the greater error at the basal levels. In rats, a small peak of hydrogen excretion was observed at 2–3 h after administration of purified RG or HRG and the peak value was greater for HRG than for purified RG. The results demonstrate that hydrogen excretion depends on the content of nondigestible oligosaccharides, because nondigestible oligosaccharides are more abundant in HRG than in purified RG, from which mono- and di-saccharides have been removed. Furthermore, this suggests that the amount of nondigestible oligosaccharides is very little compared with the amount of carbohydrate of high molecular weight in purified RG and HRG. In contrast, the production of hydrogen was maintained for a long time, because FOS is readily and completely fermented by intestinal microbes existed abundantly in the gastrointestinal tract after the administration of FOS. If high molecular weight carbohydrates in RG and HRG are fermented spontaneously by intestinal microbes, hydrogen must be excreted actively as much as FOS and would not decrease within 6-8 h after administration. These results suggest that purified RG and HRG contain a small amount of fermentable oligosaccharides and a large amount of high molecular weight carbohydrates which are resistant to fermentation.

Purified RG and HRG are partially hydrolyzed by small intestinal enzymes and are lightly fermented by intestinal microbes in rats [7]. In particular, high molecular weight carbohydrates, which are the main component of RG and HRG, were hardly hydrolyzed by small intestinal enzyme and were scarcely fermented by intestinal microbes. Therefore, a large proportion of RG and HRG would be excreted in the feces. However, the recovery for 24 h after administration to rats was very low. They were 23.4 % for purified RG and 20.2 % for HRG. One reason for this is that the perfect recovery of feces is very difficult, because the stool becomes moistened with urine adhering to the Metabolica apparatus. Another reason is that a part of administered RG and HRG may remain in the gastrointestinal tract, because the collection period (24 h) for cecal and colonic contents and stools might have been too short. In addition, the extraction of RG and HRG from stools might not have been complete. However, it could be confirmed that RG and HRG are excreted into feces.

The results obtained from the experiments using rats have demonstrated that purified RG, which the content of dietary fiber is 99 %, is more resistant to digestion and fermentation than HRG, which the content of dietary fiber is 82 %. A part of oligosaccharide fractions, which are the minor components of RG and HRG, is digested by small intestinal enzymes, and other oligosaccharide fractions, which are not digested by small intestinal enzymes, are fermented by intestinal microbes. It has also shown that high molecular weight carbohydrates, which are the main component of RG and HRG, are difficult to be digested and fermented.

### Human experiments using healthy subjects

The blood glucose and insulin levels increased slightly but obviously after oral administration of purified RG or HRG (400 mg) in rats (Fig. 2). However, they increased scarcely after ingestion of purified RG (30 g) or HRG (30 g) in the experiments using human participants (Fig. 4). This discrepancy could be explained partially by the observation that the hydrolysis of purified RG and HRG was slightly lower in humans than in rats in the previous experiments using small intestinal homogenates [7]. The similar phenomenon, which the hydrolyzing activity is lower in humans than in rats, has also been observed for highly cross-linked starch [31]. However, the hydrolyzing activity for sucrose and maltose, which are consisted of a simple structure, is similar between humans and rats [32]. The hydrolyzing activity for carbohydrates such as RG and HRG with a unique property may be different between humans and rats. The response of blood glucose and insulin by ingesting RG and HRG seem to be different in humans when composed to rats. In addition, since the dosage of the test substance per kg of body weight is calculated as 1333 mg for rats and 545-588 mg for humans, this difference may have also brought the low response of blood glucose in humans.

The content of dietary fiber, which is resistant to hydrolyzing enzymes in AOAC 2001.03 method [8], is 99 % for purified RG and 82 % for HRG. Thus, 1 % of purified RG and 18 % of HRG are digestible saccharides producing 4 kcal/g. A part of dietary fiber fraction in RG and HRG is fermented by intestinal microbes and hydrogen is produced. In humans, the breath hydrogen excretion after HRG ingestion was higher than that for purified RG, but not statistically significant. These results demonstrate that HRG contains more fermentable oligosaccharides compared with purified RG, although the dietary fiber content is less than purified RG. But, the excretion of breath hydrogen after HRG ingestion (30 g) was very low compared with that after ingesting of fermentable FOS (5 g). In addition, these results were similar to those observed in rats. If high molecular weight carbohydrates in RG and HRG are readily fermented by intestinal microbes, the excretion of breath hydrogen must be greatly enhanced and maintained the high level until the end of the experiment as well as FOS. Moreover, the hydrogen excretion by ingesting RG and HRG indicates that short chain fatty acids, which are further metabolized to energy in the host, are produced by intestinal microbes. Therefore, the available energy which is brought by the fermentation of RG and HRG is not 0 kcal/g. However, in the present study the available energy including that of digestible saccharides was evaluated as 0 kcal/g for purified RG and 1 kcal/g for HRG, respectively.

The available energy calculated from fermentation of nondigestible saccharide and available saccharide was 0.18 kcal/g (0.14 + 0.04 = 0.18) for purified RG, which the dietary fiber content is 99 %, and was 0.93 kcal/g (0.21 + 0.72 = 0.93) for HRG, which is more fermentable than purified RG and the dietary fiber content is 82 %. The hydrogen excretion showed only a small peak at 3 h after ingestion of purified RG or HRG (400 mg/2.5 ml) in rats. But, the pattern of hydrogen excretion after RMD administration (400 mg/2.5 ml) was clearly different from those after administration of RG and HRG. In other word, the hydrogen excretion of RMD showed a small shoulder at 3 h and a maximum excretion at 10 h after administration. Furthermore, in humans a peak of breath hydrogen is also showed at 6-10 h after ingestion of RMD as well as rats [5]. The high molecular weight carbohydrates, which are contained in RMD seem to be fermented more readily than those in RG and HRG in rats. Hence, the available energy of RMD, which the content of digestible oligosaccharides is less than HRG, is estimated as 1 kcal/g by our proposed method [5] and other different methods [14].

Extensive intake of nondigestible and/or non-absorbable mono- or oligo-saccharides such as FOS, 1-kestose, galactosylsucrose, lactulose, trehalose, cellobiose, lactitol, xylitol and D-tagatose induces essentially loose stool and abdominal symptoms such as thirst, flatus, distension or borborygmus in rats and humans, and each maximum non-effective dosage on them are evaluated using healthy human subjects [25–28, 33–35]. But, natural dietary fiber materials with high molecular weight do not cause osmotic loose stool. Abdominal symptoms and loose stool are influenced by the rate of digestibility and absorbability, molecular weight and habituation of sugar substitutes. The maximum non-effective dose of nondigestible oligosaccharides is 0.3-0.4 g/kg of body weight [6, 25]. Since RG and HRG contain the small amount of low molecular weight saccharides which are resistant for digestion, the extensive intake of them may induce abdominal symptoms and loose stool. But, none of the participants experienced loose stools and abdominal symptoms after ingesting 30 g of purified RG or HRG. However, two participants experienced light loose stools, flatus, and borborygmus in the preliminary experiment after ingesting 50 g of HRG (data not shown). These results suggest that ingesting 30 g of purified RG and HRG does not induce side effects in healthy adult.

On the other hand, FDA (Administration of Drug and Food) in USA does not decide the maximum non-effective dose of PD and RMD which are similar characteristics to RG and HRG and contain much small molecular nondigestible saccharides; in spite of they cause more readily loose stool than RG and HRG [36]. Side effects such as abdominal symptoms and loose stool may not be important to dietary fiber ingredient which the intake is much in quantity under the law. But, the maximum non-effective dose for abdominal symptoms and loose stool is essential for design of processed foods. Therefore, it is important knowledge that the non-effective dose of RG or HRG as ingredients is more than 30 g, which are not ingested once in the common living life. However, the ingestion of 30 g of purified RG and HRG may induce side effects in children and aged persons. Further studies are necessary for children, aged persons and pregnant women.

In previous experiment using rats, the body weight gain was normal in all groups given the test diet or the experimental diet, and the blood biochemical parameters were not significantly different among the control, RG and HRG groups in consecutive feeding of rats using diets containing purified RG or HRG [7]. Thus, RG and HRG did not harm the growth and development of rats. Furthermore, side effects such as loose stool and abdominal symptoms do not appear after ingestion of RG and HRG (30 g) in healthy adults. In addition, the abnormality was not detected in acute, subacute and chronic toxicity tests of RG [1, 9]. Therefore, RG and HRG can be used aggressively in processed foods as a dietary fiber supplement.

The indication of energy is an essential item in the nutritional labelling for processed foods. The available energy of purified RG and HRG was evaluated according to the indirect and simple method in the present study. This method has been proposed by the Japanese Society for Dietary Fiber Research, with the relative available energy of newly developed dietary fiber and nondigestible oligosaccharide materials being evaluated using FOS as a convenient reference [4, 5, 25]. The breath hydrogen excretion is used as an indicator of extent of fermentation in the proposed method to evaluate the available energy, and FOS, which the available energy is 2 kcal/g in practice, is used as a reference of typical nondigestible and fermentable oligosaccharide [4, 5]. The expiration to measure the hydrogen needs to collect for 14 h or longer after administration of test substances, because some test materials are slowly fermented by intestinal microbes in humans [5]. The collection period for more than 14 h is given more stress for subjects. The available energy is calculated from the AUC of breath hydrogen excretion for 14 h against that of FOS.

In this method, 5 g of the test substance was ingested by healthy participants, and the expiration was collected to measure the hydrogen excretion for 14 h after ingestion. The AUC of breath hydrogen excretion for 14 h after 5 g ingestion of test substances were 11,740 ppm for FOS, 850 ppm for purified RG and 1250 ppm for HRG, respectively. The available energy from fermentation is calculated as 0.14 kcal/g for purified RG and 0.21 kcal/g for HRG, because the value of FOS, which is fermented completely by intestinal microbes, is 2 kcal/g. The content of dietary fiber, which is resistant for hydrolyzing enzymes in AOAC 2003.01method, is 99 % for purified RG and 82 % for HRG, respectively. Since 1 % of purified RG and 18 % of HRG are digested and converted to energy production, the available energy becomes 0.04 kcal/g for purified RG and 0.72 kcal/g for HRG. Therefore, total available energy of digestible and nondigestible saccharides becomes to 0.18 (0.14 + 0.04 = 0.18) kcal/g for purified RG and 0.93 (0.21 + 0.72 = 0.93) kcal/g for HRG. The practical nearest whole number after rounding is 0 kcal/g for purified RG and 1 kcal/g for HRG. The difference of available energy is dependent on the difference of dietary fiber content in purified RG and HRG.

## Conclusions

The ingestion of purified RG or HRG (30 g) increased scarcely blood glucose and insulin levels and excreted slightly hydrogen in healthy human subjects. A small amount of oligosaccharides in RG and HRG were only digested and fermented slightly, while a large amount of high molecular weight carbohydrates were digested scarcely and fermented barely in healthy humans. Therefore, the bioavailability of purified RG and HRG was very low in both humans and rats, although the response of blood glucose and insulin, and breath hydrogen excretion were slightly different between humans and rats. Moreover, the ingestion of 30 g of RG or HRG did not induce any apparent side effects in healthy adults. Available energy was evaluated as 0 kcal/g for purified RG and 1 kcal/g for HRG. Therefore, RG and HRG could be potentially used as new dietary fiber materials with low energy.

## Abbreviations

RD: Resistant glucan
HRD: Hydrogenated resistant glucan
HPLC: High performance liquid chromatography
AOAC: The Association of Official Analytical Chemists
FOS: Fructooligosaccharide
AUC: Area under the curve
RMD: Resistant maltodextrin
PD: Polydextrose
AST: Aspartate transaminase
ALT: Alanine transaminase

## References

[1] Hamaguchi N, Hirai H, Aizawa K, Takada M (2014). Production of water-soluble indigestible polysaccharides using activated carbon. J Appl Glycosci.

[2] The Ministry of Health and Labour and welfare. Detailed enforcement regulations for the application of Japanese Health Promotion Law.1995. http://www.mhlw.go.jp/ to www.mhlw.go.jp/shingi/2004/12/dl/s1202-4g.pdf. Accessed 25 December, 2015.

[3] U.S. Department of Health and Human Services, Food and Drug Administration, Center for Food Safety and Applied Nutrition (2013). A Food Labeling Guide - Guidance for Industry.

[4] Nakamura S, Oku T (2005). Evaluation of available energy of several dietary fiber materials based on the fermentability from breath hydrogen excretion in healthy human subjects. J Jpn Assoc Dietary Fiber Res.

[5] Oku T, Nakamura S (2014). Evaluation of the relative available energy of several dietary fiber preparations using breath hydrogen evolution in healthy humans. J Nutr Sci Vitaminol.

[6] Oku T, Nakamura S (2002). Digestion, absorption, fermentation and metabolism of functional sugar substitutes and their available energy. Pure ApplChem.

[7] Oku T, Tanabe K, Morita S, Hamaguchi N, Shimura F, Nakamura S (2015). Digestibility of new dietary fiber materials, resistant glucan and hydrogenated resistant glucan, and physical effects by consecutive feeding in rats. Br J Nutr.

[8] Joint FAO/WHO Food Standards Programme (2010). Codex Committee on Nutrition and Foods for Special Dietary Uses 32th Session. Draft table of Conditions for Methods of Analysis for Dietary Fiber.

[9] Bito H, Hamaguchi N, Hirai H, Ogawa K. Safety evaluation of newly-developed dietary fiber: resistant glucan mixture. J Toxicol Sci, 2016; 41 (in press). 10.2131/jts.41.33 26763391

[10] Hashizume C, Kishimoto Y, Kanamori S, Yamamoto T, Okuma K, Yamamoto K (2012). Improvement effect of resistant maltodextrin in humans with metabolic syndrome by continuous administration. J Nutr Sci Vitaminol.

[11] Bear DJ, Stote KS, Henderson T, Paul DR, Okuma K, Tagami H (2014). The metabolizable energy of dietary maltodextrin is variable and alters fecal microbiota composition in adult men. J Nutr.

[12] Auerbach MH, Craig SAS, Howlett JF, Hayes KC (2007). Caloric availability of polydextrose. Nutr Rev.

[13] Konings E, Schoffelen PF, Stegen J, Blaak EE (2013). Effect of polydextrose and soluble maize fiber on energy metabolism, metabolic profile and appetite control in overweight men and women. Br J Nutr.

[14] Goda T, Kajima Y, Suruga K, Tagami H, Livesey G (2006). Availability, fermentability, and energy value of resistant maltodextrin: modelling of short-term indirect calorimetric measurements in healthy adults. Am J Clin Nutr.

[15] Oku T, Yamada K, Kanaya K (2002). Estimation of available energy of dietary fiber in various food materials. J Jpn Assoc Dietary Fiber Res.

[16] Oku T, Aoe S, Kanaya K, Kurasawa S, Sanada H, Yamada K (2013). Opinion of evaluation of available energy on Luminacoid (dietary fiber), and available energy of methylcellulose, hydrogenated resistant maltodextrin and high cross starch by the method. J Jpn Assoc Dietary Fiber Res.

[17] Oku T, Tokunaga T, Hosoya N (1984). Nondigestibility of a new sweetener, “Neosugar” in the rat. J Nutr.

[18] Tokunaga T, Oku T, Hosoya N (1989). Utilization and excretion of a new sweetener, fructooligosaccharide (Neosugar), in rats. J Nutr.

[19] Reeves PG, Nielsen FH, Fahey GC (1993). AIN-93 purified diets for laboratory rodents: final report of the American Institute of Nutrition ad hoc writing committee on the reformulation of the AIN-76A rodent diet. J Nutr.

[20] Oku T, Murata-Takenosita Y, Yamazaki Y, Nakamura S (2014). D-sorbose inhibits disaccharidaseactivity and demonstrates suppressive action on postprandial blood levels of glucose and insulin in the rat. Nutr Res.

[21] Trinder P (1969). Determination of blood glucose using an oxidase-peroxidase system with a non-carcinogenic chromogen. J Clin Pathol.

[22] Liversy JH, Hodgkinson SC, Round HR, Donald RA (1980). Effect of time, temperature and freezing on the stability of immunoreactive LH, FSH, TSH, growth hormone, prolactin and insulin in plasma. Clin Biochem.

[23] Hongo R, Nakamura S, Oku T (2010). Utilization of orally administered D-[^14^C] mannitol via fermentation by intestinal microbes in rats. J Nutr Sci Vitaminol.

[24] Tanabe K, Nakamura S, Oku T (2011). Fatal imperfection of enzymatic-HPLC quantitative HPLC method for non-digestible oligosaccharides and its proposed solution strategy. Current Nutr Food Sci.

[25] Oku T, Nakamura M, Hashiguchi-Ishiguro M, Tanabe K, Nakamura S (2009). Bioavailability and laxative threshold of 1-kestose in human adults. Dynamic Biochem Process Biotech Mol Bio.

[26] Oku T, Okazaki M (1999). Effect of single and divided ingestions of the nondigestible oligosaccharide “galactosylsucrose” on transit diarrhea and laxative threshold in normal female subjects. J Jpn Soc Nutr Food Sci.

[27] Oku T, Nakamura S, Ichinose M (2005). Maximum permissive dosage of lactose and lactitol for transitory diarrhea, and utilizable capacity for lactose in Japanese female adults. J Nutr Sci Vitaminol.

[28] Yamazaki Y, Nakamura S, Shimura F, Oku T (2011). Maximum permissive dosage for transitory diarrhea, estimation of available energy, and fate of D-tagatose in healthy female subjects. J Jpn Soc Nutr Food Sci.

[29] Oku T, Nakamura S (2003). Comparison of digestibility and breath hydrogen gas excretion of fructooligosaccharide, galactosylsucrose, and isomaltooligosaccharide in healthy human subjects. Eur J Clin Nutr.

[30] Nakamura S, Oku T (2002). Replicability of the effect of galactosylsucrose-containing food for specified health uses on fecal improvement in the case of availableness on usual life. J Jpn Assoc Dietary Fiber Res.

[31] Tachibe M, Ohga H, Nishibata T, Ebihara K (2011). Digestibility, fermentability, and energy value of highly cross-linked phosphate tapioca starch in men. J Food Sci.

[32] Oku T, Tanabe K, Ogawa S, Sadamori N, Nakamura S (2011). Similarity of hydrolyzing activity of human and rat small intestinal disaccharidases. Clin Experi Gastroenterol.

[33] Nakamura S, Ichinose M, Oku T (2004). Bioavailability of cellobiose by tolerance test and breath hydrogen excretion in humans. Nutrition.

[34] Oku T, Okazaki M (1998). Transitory laxative threshold of trehalose and lactulose in healthy women. J Nutr Sci Vitaminol.

[35] Oku T, Nakamura S (2006). Threshold for transitory diarrhea induced by ingestion of xylitol and lactitol in young male and female adults. J Nutr Sci Vitaminol.

[36] Oku T, Okamatsu H, Fujii Y (2000). Effect of polydextrose on fecal weight and gastrointestinal transit time in beagle dogs. J Jpn Assoc Dietary fibre Res.

